# Global Governance for Improved Human, Animal, and Planetary Health: The Essential Role of Schools and Programs of Public Health

**DOI:** 10.3389/phrs.2021.1604610

**Published:** 2021-12-13

**Authors:** John Middleton, Dorothy Biberman, Laura Magana, Rocio Saenz, Wah Yun Low, Philip Adongo, Gregory S. Kolt, Rajendra Surenthirakumaran

**Affiliations:** ^1^ Association of Schools of Public Health in the European Region, Brussels, Belgium; ^2^ Association of Schools and Programs of Public Health, Washington, DC, United States; ^3^ Alianza Latinoamericana de Salud Global, Santiago De, Chile; ^4^ Asia-Pacific Academic Consortium for Public Health, Kuala Lumpur, Malaysia; ^5^ Association of Schools of Public Health Africa, Accra, Ghana; ^6^ Council of Academic Public Health Institutions, Deakin, ACT, Australia; ^7^ South East Asia Public Health Institutions Network, University of Jaipur, Jaipur, India

**Keywords:** COVID-19 pandemic, preparedness and response plans, global governance for health, essential role fo schools of public health, investment in training and education, professionalisation of public health, digital public health competencies

**Statement of the Global Network for Academic Public Health (including ASPHER)**

## About the Global Network for Academic Public Health

The Global Network for Academic Public Health (GNAPH) is an alliance of regional associations that represent schools and programs of public health around the world. GNAPH is focused on enhancing academic public health worldwide through mutual learning and collaborations between academic public health institutions globally to improve and protect the health of people and the planet [1].

## Background

Since March 2021, the world’s leaders have expressed a desire to “build back better,” reflecting a desire to see an enhanced post-pandemic world and the need to improve and protect health by being better prepared to respond to future pandemics. On March 30, 2021, 25 of the world’s leaders signed an accord calling for a new pandemic preparedness treaty [2]. There is great complexity in achieving an international health treaty [3]. The Independent Panel for Pandemic Preparedness and Response, appointed by the World Health Organization (WHO), reported on May 14, 2021 [4]. They called for better funding for WHO, a Global Council for Health threats to work collaboratively with the WHO, and the delivery of the global vaccine program. They called for “21st century health data surveillance” and transparency in data sharing across countries. The Rome Declaration of the Global Health Summit of the G20 [5], called for better preparedness, for support to low- and middle-income countries and for better global financing and governance for public health and health care. The Carbis Bay Declarations of the G7 governments set out proposals for a better planet, economy, and health [6]. The European Union has set out plans to strengthen its capabilities through a Health Emergency Responsiveness Agency (HERA) [7]. The vision for HERA may be an example of how other supra-national, continental, or WHO regional health response agencies could be organized and resourced [8]. A Special Session of the World Health Assembly which took place in November 2021 has agreed to set up an Intergovernmental negotiating body to move forward the process of establishing a new Global Pandemic Preparedness Treaty [9].

## The Role of Schools and Programs of Public Health in the Current Pandemic and in Developing Standards and Competence for Better Public Health

Global leaders’ aspirations for better surveillance, greater response capacity, better global preparedness and preventive skills, better governance, and interdisciplinary collaboration must be translated into practical learning and professional development [10]. Schools and programs of public health have extensive expertise in delivering education and training as well as building capacity for the many roles that will require public health professionals in the future.

We set out some of the current actions and programs of the GNAPH member associations of schools and programs of public health around the world, which are contributing to public health training, education, workforce capacity building, and professional development in our extended paper [11]. Many of our associations’ members are providing real-time, on-the-ground support for the public health pandemic response through epidemiological input, data modelling, intervention roll-out, and health promotion activities. These programs range from implementation phase to advanced stage of development and implementation. All will require continued and reliable investment by international and national funding agencies, to secure extensive roll out, to protect and improve the health of people and the planet.

An important role of academic public health institutions is to serve our local, national, and global communities—from educating journalists and the public about important public health issues to collaborating with health departments on just-in-time trainings to providing expert guidance for decision-makers in government, non-profit, and for-profit sectors. Indeed, the public health experts on whom we have all come to rely during the pandemic were educated by, and work for and with, schools and programs of public health around the world.

Major investment is required in a professional expert public health workforce. We set out some of the actions which schools and programs of public health are uniquely placed to deliver if the goal of better health is to be achieved. Delivering better health requires trust [12] between governments and public health experts, and between them and the people and communities they serve, however defined. Moreover, we believe there should be a strong expectation of action from schools and programs of public health by our governments, from international agencies, and from the public. This should form the basis of an accord, or compact, with schools and programs of public health, reflecting their commitment to serve their populations honestly and with integrity, applying the best science available.

## Public Health Knowledge and Skills Shown by the Pandemic to Need Massive Expansion

The COVID-19 pandemic has, in many cases, put public health professionals in the public leadership spotlight, and has demonstrated the need for them to develop skills beyond the traditional public health education domains (e.g., epidemiology, biostatistics, health promotion, health policy, program evaluation) to be most effective in responding to crises. The public health workforce needs to develop new competencies that allow for multidisciplinary communication and collaboration as well as staying up to date on the traditional skills through professional development activities.

For public health professionals, essential skills include:

1. Leadership, especially of multidisciplinary groups and for multiple interests
2. Partnership and collaboration
3. Communication, particularly during crisis situations [13–16]
4. Networking with other key disciplines impacting on the health of the public including climate science, ecology, agriculture, economics, trade, international law, political science, journalism/communication, regulatory science, and theology [13, 15–17].
5. Operating and collaborating in the political environment [14, 15]
6. Application of public health law and ethical principles [13, 15]
7. Awareness of wider global health considerations, e.g., One Health principles, climate change, economic inequities, trade and health [13, 18, 19]
8. Familiarity and comfort with digital technology [13, 15, 20]
9. Enhancing the effectiveness of responses to inequities in health, meeting the needs of vulnerable, marginalized, and disadvantaged communities, indigenous peoples, refugees and migrants [13, 15, 21]
10. Refresher trainings in core public health competencies in infectious disease, epidemiology, health promotion, surveillance, vaccinology, evaluation of healthcare technologies [13, 21]

Decision-makers from government and across all fields also need to establish at least basic knowledge in public health issues to understand and best serve their communities in preventing and responding to public health crises, adapting health services to meet urgent needs, and ensuring inequities are not deepened.

For decision-makers:

1. Basic level understanding of public health values and ethics [13, 16]
2. Basic level understanding of epidemiological and other public health technical knowledge [13]
3. Basic level understanding of preparedness and response strategies, particularly processes in their region [14, 15]
4. Knowledge of available public health resources and authorities.
5. Understanding of how health-impacting events happening outside of a country or community affect the health of their population [14, 15].
6. Understanding of how laws and policies in other areas (e.g., housing, agriculture, land use, etc.) can impact the health of their population [15]
7. Basic understanding of the principles of One Health, recognizing the connection between the health of people, animals, and the environment [13, 18].
8. How to use data and evidence for decision making [15, 20].
9. How to communicate effectively with populations [13, 15, 21]
10. How to build consensus and act collectively [15]

## Declaration of the Global Network for Academic Public Health

We, the Global Network for Academic Public Health, GNAPH, representing the schools and programs of public health in our regions, believe better global governance for health requires:

1. A collective global vision and the requisite political will to deliver healthier people, animals, and planet.
2. Recognition of health as a human right for all populations.
3. A collective global commitment to transparency in all actions required for public health protection, including information sharing and early warning of new threats.
4. Ensuring global health protection structures are respected and reinforced and their recommendations are acted on.
5. Investing in global health infrastructure including advanced digital surveillance for disease, infection, chemical, biological, nuclear, and environmental threats. This means investing appropriately in the WHO and appropriate supporting structures (HQ and Regions) and supporting national and regional capabilities for innovation and production of essential products and supplies.
6. Substantial investment in public health education and research establishments, if the capacity to protect and improve health is to be delivered.
7. Resourcing and mandating WHO Regional or continental supra-national agencies that can respond to health threats which cannot be addressed by single countries acting independently and unilaterally [8].
8. Addressing the extreme inequities in health status and outcomes within and between countries and regions related to the social determinants of health.
9. Commitment to reviewing international and national laws related to public health to develop the body of public health law globally to better protect people and planet.
10. Commitment to preventing pandemics, not just responding to them, by addressing the major driver of introduction of novel infectious diseases—increased contact between humans and animals caused by destruction of animals’ natural habitats by human activities.

In order to deliver the aspirations of global leaders for better health, for a better response to the current pandemic and better preparedness for future pandemics, the Global Network of Academic Public Health believes:

1. There must be increased understanding and recognition of the unique role of public health professionals, including recognition by governments and health systems for the profession of public health and for professional codes of conduct.
2. There must be a global willingness for public health professionals to be able to work across jurisdictional boundaries, with mutual recognition of qualifications.
3. There must be a strengthening of the public health profession, systems, data, and services in all countries.
4. There must be recognition of the multidisciplinary nature of the public health profession, systems, services, and all activities related to health improvement and protection.
5. The strengthening of public health systems and services will require research into workforce capacity, skills, and knowledge, and job and career opportunities at local, national, and global levels. The gaps in workforce capacity and skills identified must be addressed by investment in schools and programs of public health and other academic centers to grow the new public health workforce for local, national, and international deployment.
6. Necessary improvements in public health capacity require rapid investment in high volume, short course training for public health and other health professionals in preparedness, surveillance, and emergency response. There should be basic public health knowledge and awareness-raising training for others in related fields affecting the public’s health including economics, finance, law, trade, and journalism; for political leaders and their advisors; and the public.
7. There must be increased understanding of public health amongst other disciplines, political decision-makers, the media, and the public.
8. There must be investment in education and training for lawyers and public health experts to develop the body of national, regional, and international public health laws and regulations.
9. There must be investment in public health leadership training to develop a cadre of new leaders at local, national, and global levels capable of conveying public health knowledge, building partnerships across agencies and with the public, and organizing the action required to protect and improve the public’s health.
10. These requirements can only be met by recognizing the key role of, and investing in, schools and programs of public health as leaders in education, training, and research.

We, the leaders of the Global Network for Academic Public Health present this Declaration and related papers for consideration by our members, partners, and colleagues. We also present it for a wider audience of decision makers and funding bodies at global, regional, and national levels. We hope our member schools and programs of public health will use the declaration in their evidence to national governments, and health and education ministries, to make the case for enhanced public health education. We respectfully call on the new intergovernmental negotiating body (9) to ensure that there is a clear and substantial commitment to investment in education and training for public health built into the new treaty.
